# Differential susceptibility of C57BL/6NCr and B6.Cg-Ptprc^a ^mice to commensal bacteria after whole body irradiation in translational bone marrow transplant studies

**DOI:** 10.1186/1479-5876-6-10

**Published:** 2008-02-28

**Authors:** Raimon Duran-Struuck, Adam Hartigan, Shawn G Clouthier, Melissa C Dyson, Kathi Lowler, Erin Gatza, Isao Tawara, Tomomi Toubai, Elisabeth Weisiger, Kelly Hugunin, Pavan Reddy, John E Wilkinson

**Affiliations:** 1 Unit for Laboratory Animal Medicine, University of Michigan Medical School, Ann Arbor, MI, USA; 2 Department of Internal Medicine, Bone Marrow Transplant Section, University of Michigan Medical School, Ann Arbor, MI, USA

## Abstract

**Background:**

The mouse is an important and widely utilized animal model for bone marrow transplant (BMT) translational studies. Here, we document the course of an unexpected increase in mortality of congenic mice that underwent BMT.

**Methods:**

Thirty five BMTs were analyzed for survival differences utilizing the Log Rank test. Affected animals were evaluated by physical examination, necropsy, histopathology, serology for antibodies to infectious disease, and bacterial cultures.

**Results:**

Severe bacteremia was identified as the main cause of death. Gastrointestinal (GI) damage was observed in histopathology. The bacteremia was most likely caused by the translocation of bacteria from the GI tract and immunosuppression caused by the myeloablative irradiation. Variability in groups of animals affected was caused by increased levels of gamma and X-ray radiation and the differing sensitivity of the two nearly genetically identical mouse strains used in the studies.

**Conclusion:**

Our retrospective analysis of thirty five murine BMTs performed in three different laboratories, identified C57BL/6NCr (Ly5.1) as being more radiation sensitive than B6.Cg-Ptprc^a^/NCr (Ly5.2). This is the first report documenting a measurable difference in radiation sensitivity and its effects between an inbred strain of mice and its congenic counterpart eventually succumbing to sepsis after BMT.

## 1. Introduction

Bone marrow transplantation (BMT) is a common therapy for many neoplastic and non-neoplastic conditions [1]. Current research is focused on the development of more efficient transplant regimens and therapies [1]. Before receiving a donor bone marrow (BM) graft, BMT patients undergo preparatory regimens (which often include irradiation) to deplete the host BM. Complications of BMT include viral, bacterial or fungal infections secondary to bone marrow ablation, and relapse of neoplastic disease [2,3]. Few human patients die directly from the preparatory regimens (chemotherapy, radiation) because of the availability of supportive care. However, debilitated high risk transplant patients require careful titration of the myeloablative therapy to prevent multiorgan disease [4,5]. Some of these complications are related to failure of engraftment. One of the most deleterious side effects of allogeneic BMT (when donor and recipient are genetically different) is graft-versus-host disease (GVHD) [6]. Grafted donor cells recognize the host (recipient of BMT) tissues as foreign leading to GVHD. Acute GVHD is defined as a disease appearing within 100 days post-BMT which targets the skin, liver, lung, thymus and secondary lymphoid tissues [7].

The mouse is one of the most utilized animal models for translational BMT studies. With the development of inbred strains of mice, many discoveries in the field of immunology, immunogenetics, and radiation oncology have taken place [8]. Inbred strains of mice permit the study of BMT in genetically identical and genetically variable systems. Transplantation between animals within the same strain allow for modeling of autotransplants or transplants between genetically identical twins (syngeneic). Genetically different donor and recipient strains allow modeling of transplantation between genetically different individuals (allogeneic). The mice in this report came from a number of BMT studies conducted by three laboratories where they utilize inbred strains for both syngeneic and allogeneic BMTs. There are three groups of BMT recipient per experiment: 1) syngeneic bone marrow recipients (syngenic BMTs) in which all animals are expected to survive. 2) allogeneic bone marrow recipients (allogenic BMTs) in which animals are expected to eventually succumb to GVHD, and 3) experimental allogenic transplant recipients (experimental transplants) which receive allogeneic bone marrow with genetically induced modifications or treatments thought to prevent or decrease GVHD. Experimental animals are expected to have improved survival compared to the allogeneic control groups.

In general, mice irradiated for BMT studies experience lethargy and malaise of one week duration due to radiation induced multisystem organ damage. Commonly, all animals, whether syngeneic or allogeneic survive the post-irradiation period. Syngeneic animals usually recover within two weeks, while allogeneic groups experience a biphasic illness. After the first week of irradiation sickness, allogeneic recipients undergo a relatively short recovery period prior to becoming sick for the second time due to GVHD. Animals developing GVHD, die as early as 12–14 days post-BMT and as late as 30–40 days post-BMT depending on the degree of genetic mismatch. Mice developing GVHD may exhibit diarrhea, skin lesions (ulcerations) and overall lethargy and anorexia as a consequence of the damage to the classical GVHD target organs (skin, gastrointestinal tract, liver, thymus) [9] similar to man.

In our studies, we observed an unexpected increase in mortality very early after transplantation. One of the most striking findings was facial swelling in the affected animals. Premature deaths in experimental murine models of BMT cause significant complications in interpretation of the experimental results and delay it's translation to the clinic. Furthermore, it greatly increases expense due to the necessity of using large numbers of mice, as well as the effort of the investigators. These economic factors, the impact on experiment outcomes, and the humane considerations all indicate the need to minimize premature deaths and better understand the limitations of the animal models utilized for human research. In our studies because of the difference in the non-GVHD related early mouse deaths in both syngeneic and allogeneic BMTs, we carefully evaluated the environmental, genetic, treatment, and pathologic factors associated with early death in a large group of BM transplanted mice.

## 2. Materials and methods

### 2.1 Mice

The strains of mice used as recipients in the discussed BMT studies were; C57BL/6NCr referred to as Ly5.1 and B6.Cg-Ptprc^a^/NCr, commercially available as B6-LY5.2/Cr, and referred throughout this manuscript as Ly5.2. The specific background strain from which the Ly5.2 gene comes from is unknown. There has been considerable confusion concerning the correct nomenclature for alleles of the protein-tyrosine phosphatase, receptor type c locus (Ptprc), formerly Ly5. Ly5.1 and Ly5.2 nomenclature is still commonly utilized in transplantation. The nomenclature used in this manuscript is consistent with The Jackson Laboratory (Jax) mouse genome informatics and Charles River Laboratories. Animals were housed in AAALAC accredited specific pathogen free rodent facilities at the University of Michigan in non-sterilized, ventilated racks and supplied with commercial rodent food. Animals received tap water from municipal sources until bone marrow transplantation at which time they received acidified water, pH of 2.9–3.1 for 21 days. All animals were negative for all pathogens included in the assessment profile (Charles River^®^) and tested at the vendor's facility prior to shipping. These included: Ectromelia, Enzootic Diarrhea of Infant Mice, Mouse Hepatitis Virus, Mouse Parvovirus, Minute Mouse Virus, Reovirus-3, Sendai virus, Theiler's Murine Encephalomyelitis Virus, Lymphocytic Choriomeningitis virus, Adenovirus, *Mycoplasma pulmonis*, Polyoma virus, Pneumonia virus of mice, Cilia-associated Respiratory Bacillus. Animals received from the National Cancer Institute (NCI) were also negative for all bacterial, viral and protozoal diseases mentioned above and documented in their surveillance reports.

All procedures were approved by the University of Michigan's Animal Care and Use Committee.

### 2.2 Irradiation and BMT

Prior to transplantation, all animals received a myeloablative radiation (from 800–1300 cGy) given in a single dose, or split into two doses 3–4 hours apart as described previously [10,11]. The laboratories used the gamma irradiator, Gammacell 40^® ^(+/- 0.8 rad/min) MDS Nordion, Inc. (Ontario, Canada) or an X-ray electrovoltage instrument (Philips 250 kV orthovoltage unit, 4 rad/min). All animals were placed in a Raddisk^® ^(Braintree Scientific, Braintree, MA) when irradiated in the Gammacell 40. When exposed to X-rays animals were maintained in a custom made ventilated Plexiglas restraining device. The donor bone marrow recipients' immune system was reconstituted intravenously via the tail vein with 5 × 10^6 ^bone marrow cells with or without isolated splenic T cells (5 × 10^5 ^to 5 × 10^6 ^cells), total splenocytes or tumor cells given on the same day of irradiation. BMTs were performed in dedicated procedure rooms in the animal facilities (under HEPA-filtered hoods) or the laboratories (in a clean dedicated area for injection). The recipients were placed in a tail vein injector (custom made) and a maximum of 250 μL of BM inoculum was delivered per animal. Transplanted animals were housed in separate rooms from untreated mice.

### 2.3 Pathology

Severely ill animals that were identified 4–10 days post-BMT in different experiments were humanely euthanized. Organs (brain, thoracic and abdominal organs) were harvested and placed in 10% formalin for 24 hours and then 70% ethanol prior to slide preparation. The tissues were cut at 5 μm in thickness and stained with H&E prior to analysis.

### 2.4 Blood and tissue cultures

Animals were anesthetized and an intracardiac or orbital terminal phlebotomy was performed. Blood and tissue cultures of kidneys, liver, spleen, heart and lungs were aseptically obtained and routinely processed.

### 2.5 In-house serology

177 naïve mice used as recipients were assessed in-house (in addition to the health status reports from NCI and Charles River Laboratories) for evidence of 13 viruses and two bacteria using the Charles River Diagnostic Laboratories Assessment Profile (Charles River^®^) ELISA as described previously. Mice were from the same vendor and were analyzed during the same time period when premature deaths were occurring. The 177 mice analyzed (and chosen at random) belonged to the three investigators as they shared a common mouse arrival holding room.

### 2.8 Retrospective study of common factors

We evaluated factors related to: laboratory personnel, housing, BM inoculum differences, irradiation source (gamma or X-ray), irradiation dose, time of year BMTs were performed and housing location before and after transplantation.

### 2.8 Statistical analysis

Satistical analysis was performed as described [12]. The Wilcoxon rank test was used to analyze irradiation doses and survival. p < 0.05 was considered statistically significant. The Cohran-Mantel-Haenszel statistic for general association was performed to compare season of year (winter, spring, summer) and laboratory personel (between three laboratories). p < 0.05 was considered statistical significant. Odds ratios with a 95% confidence interval was performed when comparing radiation source (X-ray or Gamma irradiator), animal holding rooms, or inoculum differences. p < 0.05 considered statistically significant.

## 3. Results

### 3.1 Physical exam of affected recipients and early deaths post-BMT

**Figure 1:**
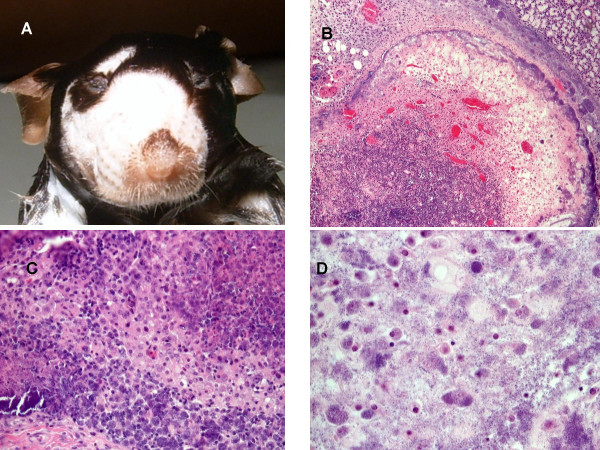
Gross examination showed marked facial and submandibular swelling (A). Histopathology of cervical (B) and mesenteric (C, D) lymph nodes showing acute, severe, necrotizing lymphdenitis and cellulitis with intralesional bacteria at 4× (B), 10× (C) and 20× (D) magnification. Acute severe necrosis (C) of mesenteric lymph node.

On physical exam affected mice were lethargic, had a hunched posture and exhibited decreased mobility. The most striking clinical sign was pronounced facial swelling (Figure 1A). In some experiments as many as 100% of the syngeneic controls died within a week (Table 1). When an experiment was affected, 25–100% of the animals would exhibit the specific clinical sign of facial swelling (Table 1).

**Table 1:** C57BL/6NCr are sensitive to irradiation doses >1000 cGy.

**Inoculum type**	**Recipients**	**First day Symptoms observed post BMT**	**#affected**	**% affected**	**Treatment**	**Rads (cGy)**	**Irradiator**
BM+ Tc	B6 (Ly5.1)	6	2/4	50		1300 split	Xray
BM + splenocytes	B6 (Ly5.1)	7	2/4	50		1100 single	Xray
BM + Tc	B6 (Ly5.1)	9	2/4	50		1100 single	Xray
BM+Tc	B6 (Ly5.1)	7	1/3	33	EL-4	1100 single	Xray
BM+Tc	B6 (Ly5.1)	7	3/12	25	EL-4	1100 single	Xray
BM+T c	B6 (Ly5.1)	6	2/6	33	EL-4	1100 single	Xray
BM+Tc	B6 (Ly5.1)	6	8/8	100	EL-4	1100 single	Xray
BM+Tc	B6 (Ly5.1)	6	11/20	55		1100 single	gamma
BM+Tc	B6 (Ly5.1)	6	2/4	50		1100 single	gamma
BM+Tc	B6 (Ly5.1)	6	4/5	80		1100 split	gamma
BM+Tc	B6 (Ly5.1)	5	4/6	66		1300 split	gamma
BM+Tc	B6 (Ly5.1)	5	9/11	81	MBL-2	1300 split	gamma
BM+DC	B6 (Ly5.1)	4	5/5	100	DC	1100 single	X-ray
BM+Tc	B6 (Ly5.1)	5	5/5	100		1100 single	gamma
BM+Tc	B6 (Ly5.1)	5	3/9	33		1000 single	gamma

C57BL/6NCr underwent BMT and exhibited unexpected deaths. Animals were reconstituted with bone marrow (BM) and T-cells (Tc), or bulk splenocytes (splenocytes) or dendritic cells (DC). Symptoms started as early as day +4 post-transplantation. Some animals received MBL-2 or EL-4 leukemia cells. Ionizing radiation was given in a single or fractionated (split) dose or administered with an X-ray diffraction instrument. Split regimens were delivered in two equal amounts separated at least 3 hours apart.

### 3.2 Systemic bacterial infection identified in affected animals on gross and histopathology analysis

Necropsy was performed on animals that exhibited facial swelling. Gross pathology revealed severe cervicothoracic swelling (Figure 1B) restricted to the dermis and subcutaneous layers. The salivary glands and the submandibular lymph nodes were often enlarged. Some cases exhibited severe swelling of the tongue.

**Figure 2:**
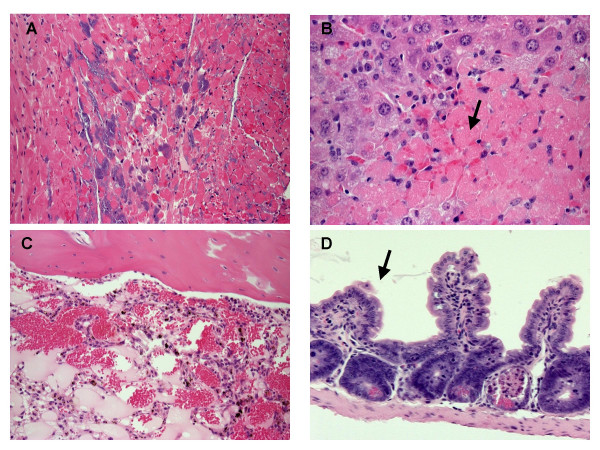
Histopathology of the heart (A), liver (B), bone marrow (C), and small intestine (D). Acute, severe bacterial myocarditis with intralesional bacteria (A) 10×. Acute hepatic necrosis (arrow) (B) 20×. Leukocyte deficient bone marrow (C) 10×. Blunted villi (arrow) in small intestine (D) 10×.

Histopathology identified an acute, severe, locally extensive, necrotizing cervical lymphadenitis and cellulitis with many intralesional bacteria (Figure 1B,C,D). There was also extensive necrosis in the liver (Figure 2B) and spleen. Numerous organs including the heart and liver contained abundant bacteria with no reaction (Figure 2A,B). Spleen, lymph nodes (Figure 1B,C,D), blood, and bone marrow (Figure 2C) were leukocyte deficient indicating a state of immunosuppression. The villi of the small Intestine were severely blunted and fused consistent with radiation induced gastrointestinal damage (Figure 2D).

### 3.3 Commensal bacteria and their toxins identified in blood and tissues

In view of the above results, bacterial cultures were analyzed from twelve affected animals exhibiting the exact same symptoms from different experiments. These cultures revealed a mix of bacteria in both blood and tissues including *Escherichia coli*, *Pseudomonas spp*., *Enterococci spp*., *Staphylococcus spp*., *and Streptococci spp*.

### 3.4 In-house serological analysis of naive animals

We aimed to rule out the possibility that naïve mice shipped from our vendors, and documented to be negative of all infectious diseases tested (described in materials and methods) did not become positive after being housed in our facilities. Therefore, 177 naïve animals (to be used in future transplants and which were housed in a common room shared by the three investigators) were tested a second time for the same pathogens utilizing the Charles River Diagnostic Laboratories Assessment Profile, (Charles River^®^) (refer to materials and methods). All animals were negative for all pathogens tested.

### 3.5 Multi-factorial analysis of BM transplanted animals

**Figure 3:**
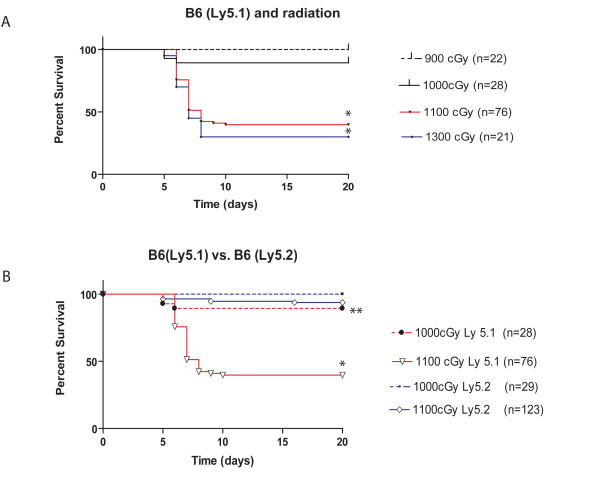
**B6 (Ly5.1) mice are more sensitive to irradiation than B6-Ly5.2**. (A) Survival comparison in the Ly5.1 mice after different irradiation doses. Ly5.1 are susceptible to irradiation doses ≥ 1100 cGy. All animals received a BMT the same day of irradiation (B). Survival comparison between congenic B6-Ly5.2 mice and Ly5.1 mice post irradiation doses of 1100 cGy, 1000 cGy or less. Ly5.2 mice are more radioresistant than Ly5.1 mice at TBI doses of 1100 cGy. *p < 0.001, **p = 0.068.

We retrospectively evaluated environmental, experimental and animal factors associated with the study including: strain of mouse, housing location, experimental equipment, the degree of irradiation, type of irradiation (gamma or X-ray), time a year (winter, spring, summer), laboratory (comparing BMTs from the three laboratories), treatment (splenocytes or tumor cells). Factors that were not common among the three laboratories involved were cell preparations, procedure rooms, housing location of transplanted animals, cell culture media, and surgical instruments. The most important factor identified in the early deaths was the dose of radiation and the genetic background of the recipient mice. Affected mice were identified to be among the C57BL/6NCr (Ly5.1) group while B6.Cg-Ptrpc^a^/NCr (Ly5.2) congenics were minimally or not affected (Table 2). Statistically significant differences in survival were found using a Wilcoxon rank test between Ly5.1 and Ly5.2 animals that received irradiation (Figure 3A,B). Increased mortality (p < 0.001) was observed in the Ly5.1 mice that received ≥ 1100 cGy (Figure 3A). The strain of the donor mice, the inclusion of T cells or splenocytes in the inoculum, and genetic compatibility of the donor and recipient did not affect morbidity and mortality. Radiation at, or under 1000 cGy in B6 Ly5.1 recipients had a significant (p < 0.001) improvement in survival (Figure 3A and Table 3). When comparing variables for survival with more than two levels (laboratory and season), the test for general association for the different laboratories had a p = 0.165 and seasonal influence had a p = 0.116. Odds ratios between differences by animal room post BMT had p value for the chi-square statistic of 0.10. Odds ratios for being more affected with X-ray compared to gamma radiation had a p = 0.35. Odds ratio for groups that did not receive neither splenocytes nor tumor cells p = 0.62. We considered statistically significance if p < 0.05.

**Table 2:** Improved survival of B6.Cg-Ptprc^a^/NCr mice to radiation doses of 1000 and 1100 cGy.

**Inoculum type**	**Recip**	**Symptoms**	**#affected**	**% affected**	**Rads (cGy)**	**Irradiator**
BM	B6 (Ly5.2)	None	0/10	0	1000 (single)	X-ray
BM	B6 (Ly5.2)	None	0/10	0	1000 (single)	X-ray
BM	B6 (Ly5.2)	None	0/10	0	1000 (single)	X-ray
BM+Tc	B6 (Ly5.2)	None	0/5	0	1100 (single)	gamma
BM+Tc	B6 (Ly5.2)	none	0/9	0	1100 (single)	gamma
BM+Tc	B6 (Ly5.2)	None	0/27	0	1100 (split)	gamma
BM +Tc	B6 (Ly5.2)	None	0/30	0	1100 (split)	gamma
BM+Tc	B6 (Ly5.2)	None	0/25	0	1100 (split)	gamma
BM+ Tc	B6 (Ly5.2)	Yes	7/24	29%	1100 single	gamma

B6.Cg-Ptprc^a^/NCr (B6LY5.2/Cr), referred as Ly5.2 congenics, receiving similar radiation doses as shown in Table 1. Recipients were reconstituted in these experiments with either bone marrow (BM) alone or BM and T-cells (Tc).

**Table 3:** C57BL/6NCr survival at doses under 1000 cGy.

**Inoculum type**	**Recipients**	**Symptoms**	**#affected**	**% affected**	**Rads (cGy)**	**Irradiator**
BM+Tc	B6 (Ly5.1)	None	0/5	0	1000 single	gamma
BM+Tc	B6 (Ly5.1)	None	0/5	0	800 split	X-ray
BM+Tc	B6 (Ly5.1)	None	0/5	0	800 split	gamma
BM+ Tc	B6 (Ly5.1)	None	0/5	0	900 single	gamma
BM+ Tc	B6 (Ly5.1)	None	0/6	0	900 single	gamma
BM+ Tc	B6 (Ly5.1)	None	0/5	0	900 single	gamma
BM+ Tc	B6 (Ly5.1)	None	0/3	0	900 single	gamma
BM+ Tc	B6 (Ly5.1)	None	0/6	0	1000 single	gamma
BM+ Tc	B6 (Ly5.1)	None	0/6	0	900 single	gamma
BM+ Tc	B6 (Ly5.1)	None	0/3	0	1000 single	gamma
BM+ Tc	B6 (Ly5.1)	None	0/5	0	1000 single	gamma

C57BL/6NCr that received ≤ 1000 cGy survived the bone marrow transplant.

## 4. Discussion

In our studies we document the clear differences in mortality between C57BL/6NCr (Ly5.1) and B6.Cg-Ptrpc^a^/NCr (Ly5.2)C57BL/6Cr mice in BMT studies that utilized irradiation as the main immunosuppressive regimen for translational transplant studies. When syngenic animals (positive controls), which would otherwise survive, started dying, this made the transplants uninterpretable. The death of immunocompromised mice such as SCIDs and Nude (nu/nu) mice are common when exposed to bacterial, viral, parasitic or protozoal pathogens similar to immunocompromised individuals [13-17]. However following BMT, despite transient immunosuppression, immunocompetent transplanted animals usually survive for greater than seven days after the immunoablative treatment [10,18-23]. If there is lack of engraftment the animals will eventually succumb to infection secondary to BM aplasia [24,25] and anemia or thrombocytopenia. Premature death may occur if the BM inoculum is not successfully delivered, or the delivered cells are unable to engraft and repopulate the hemolymphatic system of the new host. In the cases reported here, all animals were given a BMT dose and were expected to survive the initial immunosuppressive period. Failure to deliver an appropriate dose of viable cells is unlikely because the intravenous tail vein injections were given by experienced individuals and the Ly5.2 mice that were not affected were given the same dose, by the same route, and by the same personnel as those mice that died prematurely.

Animals might die early if aged, debilitated or if burdened by a subclinical infection that was not identified prior to myeloablation. This effect has also been observed in the clinic with elder or very critical patients [4]. In our studies, the mice undergoing transplantation were 3–8 months old and healthy (based on NCI's health reports). As previously mentioned, mice that receive myeloablative conditioning prior to BMT survive the time period immediately after the preparatory regimen (5–7 days post irradiation). By day +14 post-BMT, there is immunological recovery which is demonstrated by normal peripheral immune reconstitution [26]. As is expected in *in vivo *studies, some intra-animal variation may occur, however if a mortality over 20% in the syngeneic controls is observed, one must re-assess the experimental protocol. Mice receiving allogeneic BM, and T cells may die of severe GVHD after 2 weeks post-transplantation. Depending on the T cell and BM dose and the degree of mismatch, GVHD may develop before 14 days post-BMT. However, 1) syngeneic controls should survive and 2) facial swelling should not be observed as it is not a feature of GVHD in mice.

The premature animal deaths in this study were independent of the transplant details (donor T cell source, personnel, housing, cell preparation, irradiation source, age and sex of recipient or housing facilities). Some mouse viruses have been documented to be immunosuppressive and impede stem cell differentiation [27-32] possibly having an influence in immune reconstitution and engraftment. Serology of 177 naïve animals to be used at a later time as either donor or recipients, were negative for all bacteria and viruses including parvoviruses. The strain of the recipient and the dose of irradiation were the two predominant factors influencing facial swelling and mortality in these BMT models (Figure 3A,B).

We identified *E. coli*, *Pseudomonas spp., Enterococcus *and *Streptococci spp *in the blood and tissues. These pathogens have commonly been reported to affect immunocompromised animals [33]. In order to prevent infections in BMT mice some groups recommend treating the animals with antibiotics in the water in order to minimize bacterial contamination post irradiation. In our institution we utilize acidified (hyper-chlorinated) water with a pH = 2.9–3.0 [19,20] and have rarely experienced problems in mice in these study protocols.

Based on previous studies [22,23], gut damage in animals that are SPF (not germ free), have some degree of translocation of bacterial toxins (e.g. LPS) and possibly bacteria post-irradiation, which in turn causes a severe inflammatory response and severe hypotension. This is known to be mediated by TNFα and IL-1 inflammatory mediators [9]. However, and as previously mentioned, during uncomplicated bone marrow transplants, syngeneic animals recover successfully after this period. Based on the blood and tissue cultures and histopathology, we clearly document that the affected mice were severely septic. Even though death from sepsis may be expected in severely immunocompromised mice if challenged with a pathogen, early death from sepsis on transplanted animals, though possible, it is not common. In our studies, the systemic peri-BMT preparatory regimen caused a degree of damage where even syngeneic recipients were unable to successfully recover from the early post-transplant period. As documented, their deaths were likely caused by the translocation of gastrointestinal bacteria and their toxins into the circulation, and subsequently into other organ systems. Such effects continue to be an important co-morbidity factor in human BMTs [1].

Facial swelling has been documented in immunodefficient SCID mice after severe infections in their oropharyngeal regions [17,33]. Therefore, the swelling in our animals can, in part, be explained by the autoinoculation of bacteria during their vulnerable immunocompromised period as described in SCID mice.

In our studies the dose of radiation was one major factor in causing the premature deaths. It has been documented that splitting a single radiation dose into two equal half doses causes less GI damage [34]. Based on our retrospective study, we observed that only the animals that died at the early time points post-BMT (4–8 days) and exhibited facial swelling, were the mice that received the highest radiation doses performed in the laboratories (1100–1300 cGy). High radiation doses such as 1000 cGy have been documented to be relatively toxic when given to larger species such as dogs and man [35,36]. However, many strains and stocks of mice have historically been more resistant to irradiation (e.g. C3H strains) [37] while others are significantly more sensitive (e.g. BALB/c) [38] once again emphasizing the importance of genetics not only in BMT match/mismatches, but in the susceptibility to the preparatory regimens. These factors are also likely to impact human BMT outcomes.

In our retrospective analysis we have been able to identify that most of the animals affected have been C57BL/6NCr (Ly5.1) (Table 1 and Figure 3). B6.Cg-Ptrpc^a^/NCr (Ly5.2) syngeneic recipients receiving 1000–1100 cGy irradiation doses were less affected (Table 2 and Figure 3) by irradiation and the differences were statistically significant (p < 0.001). This suggests that B6-Ly5.2 congenic mice are therefore more resistant to irradiation. One group of B6-Ly5.2 mice in Table 2 exhibited a 29% mortality. It is possible that 1100 cGy given in a single dose may be the radiation limit that Ly5.2 mice can withstand in our hands. We must mention that certain variability is always observed when performing *in vivo *experiments (even in age matched and using inbred strains) and there can be unknown factors which may impact (hopefully to a lesser degree) the experimental outcomes. It is possible in the case of Ly5.2 animals mentioned above that human error calculating the irradiation dose may have influenced the outcome of this experiment, especially if we assumed that we are at the cusp of the maximal radiation dose tolerated by Ly5.2 mice. It has been our experience that Ly5.2 animals that received 1300 cGy as a single dose had a 90% mortality. In any case, and based on the number of animals included in this study, we show that Ly5.2 mice are more resistant to radiation and that no mortalities are observed when either splitting the 1100 radiation dose or decreasing the overall amount of radiation. There are a plethora of studies that have documented the link between the susceptibility of ionizing radiation and the genetic makeup [37-46] between different strains of mice and also man. For example DNA-protein kinases (DNA-Pks), which are important for DNA repair, were found to be diminished in BALB/c and 129/SvJ compared to C57BL/6 mice after being exposed to ionizing radiation [47]. In our specific study, we document for the first time a difference in sensitivity between two nearly genetically identical strains commonly used in biomedical research. Despite their nearly identical genetic makeup supported by immunological tolerance by the absence of GVHD [48] in allogeneic BMTs or rejection of skin grafts, there may still remain enough genetic disparity between the two strains to produce this observed difference in response to radiation. The mechanism of this differential sensitivity is unkown. It is possible that these two strains have differences in their DNA repair capabilities or radioprotective processes. It has been shown that the release of toxic nitric oxide radicals (NOr) can contribute to irradiation damage and that low NOr release post-irradiation is radioprotective [49]. It is also possible that Ly5.2 recipients may have decreased NOr release post irradiation compared to Ly5.1.

In conclusion, we have demonstrated for the first time a measurable difference in radiation sensitivity and its effects between two nearly genetically identical strains of mice commonly used for translational studies where C57BL/6NCr die of bacteremia more readily than B6.Cg-Ptprc^a^/NCr congenics at radiation doses over 1000 cGy.

## Abbreviations

Graft-versus-host disease (GVHD), C57BL/6NCr (B6 Ly5.1 or Ly5.1), B6.Cg-Ptprc^a^/NCr or B6-LY5.2/Cr (Ly5.2), total body irradiation (TBI)

## Authors' contributions

RDS wrote the manuscript, performed BMTs, and diagnosed all animal cases. AH performed BMTs, revised and designed manuscript and submitted pathology samples. SGC performed BMTs and participated in the design of manuscript. IT participated in the design of manuscript and performed BMTs. TT, EG performed BMTs. EW, KL revised manuscript and participated in BMTs. KH performed statistics. MD edited the manuscript and provided serologic results. PR participated in the design of the manuscript and analyzed data. JEW performed all histopathology and diagnosis and participated in the design of the manuscript. All authors read and approved the manuscript.

## Funding

NIH/NCRR T32-RR07008-21A1 (R.D.S) and Unit for Laboratory Animal Medicine internal funds.
